# Editorial: Advancements and challenges in autism and other neurodevelopmental disorders

**DOI:** 10.3389/frcha.2024.1372911

**Published:** 2024-02-12

**Authors:** Sara Calderoni, David Coghill

**Affiliations:** ^1^ Department of Developmental Neuroscience, IRCCS Stella Maris Foundation, Calambrone, Italy; ^2^ Department of Clinical and Experimental Medicine, University of Pisa, Pisa, Italy; ^3^ Faculty of Medicine, Dentistry and Health Sciences, University of Melbourne, Melbourne, VIC, Australia; ^4^ Neurodevelopment and Disability, Murdoch Children’s Research Institute, Melbourne, VIC, Australia; ^5^ Department of Mental Health, Royal Children’s Hospital, Melbourne, VIC, Australia

**Keywords:** autism spectrum disorders, neurodevelopmental disorders, children, adolescents, ADHD

**Editorial on the Research Topic** Advancements and challenges in autism and other neurodevelopmental disorders

According to the Diagnostic and Statistical Manual of Mental Disorders 5th edition (DSM-5), (1), neurodevelopmental disorders (NDDs) are a group of conditions with onset in the developmental period, inducing multiple impairments of functioning. NDDs comprise intellectual disability (ID), Communication Disorders, Autism Spectrum Disorder (ASD), Attention-Deficit/Hyperactivity Disorder (ADHD), Neurodevelopmental Motor Disorders, including Tic Disorders, and Specific Learning Disorders. In a recent systematic review, the prevalence as a whole of NDD in individuals under 18 years old is somewhere between 4.70 and 88.50%: this wide range is dependent, among other things, upon methodological factors (evaluation in clinical populations vs. the general population), and sociocontextual aspects (underdiagnosis in developing countries) (2).

The heterogeneous diagnostic categories of NDDs share a common genetic etiology (3), and are the result of altered neurodevelopmental trajectories (4). From an epidemiological and clinical point of view, NDDs are characterized by being diagnosed more often in males than females and by having high rates of comorbidities, not only among different types of NDDs, but also with other psychiatric disorders (5).

The complexity of NDDs is reflected in the wide heterogeneity of the seven papers included in this Research Topic, which explores different aspects of the various disorders within NDDs, spanning from genetic underpinnings (Wu and Li), to clinical (Alfieri et al.) and neuropsychological profiles (Donno et al., Krone et al., Krone et al.), to psychopharmacological treatment (Dimitri et al.), and finally through to psychophysiological paradigm (Pomè et al.).

Within this broad conceptual framework, the aim of the Research Topic *Advancements and Challenges in Autism and Other Neurodevelopmental Disorders* within Frontiers in Child and Adolescent Psychiatry is to promote important contributions to the field.

In the study by Wu and Li, *Genetic analysis of neurodevelopmental disorders in children,* the authors retrospectively investigate the genetic causes of NDDs in children referred to a single University hospital in China through the analyses of Chromosomal Microarray (CMA), whole exome sequencing (WES), and –only for male children- FMR1 CGG repeat analysis for fragile X syndrome. The results indicated a total of 488 (33.5%) pathogenic variations among 1,457 children with global developmental delay/intellectual disabilities and reiterate the importance of recommending genetic testing for this population: indeed, the identification of an underlying genetic etiology can provide both clinical and personal information useful to patients and their families.

Alfieri et al. present a study focussed on Malan syndrome (MALNS), an ultra-rare genetic syndrome characterized by an unusual facial phenotype, generalized overgrowth, ID and behavioral problems. In *Behavioral profiling in children and adolescents with Malan syndrome* the authors set out to shed light on the psychopathological comorbidities in a sample of 15 children and adolescents/young adults with MALNS. Results from this monocentric investigation emphasize that people with MALNS are characterized by high level of psychiatric comorbidies, particurarly anxiety and ADHD symptoms, which further complicate the global impairment and impact on adaptive functioning. The study adds to the understanding of the clinical picture of individuals with MALNS, in order to promptly recognize and treat associated psychopathology, with the ultimate goal of improving the outcome for both affected individuals and their families.

Donno et al. in *Social and executive functioning in individuals with autism spectrum disorder without intellectual disability: The case–control study protocol of the CNeSA study* described their study protocol aimed at investigating hot and cold executive functions in a cohort of 40 children and adolescents with ASD without intellectual disability compared with matched peers. Specifically, a comprehensive neuropsychological test battery, as well as an in-depth clinical assessment will be administered to participants. Moreover, autonomic measures including heart rate, heart rate variability, skin conductance, will be recorded before, during and after the neuropsychological evaluation and two salivary cortisol samples will be collected at baseline and at the end of test administration. This study protocol seeks to shed light on the complex relationship between neuropsychological/autonomic functioning and ASD symptoms in order to provide targeted intervention strategies.

Two papers from the same U.S. research group contribute to understanding in youth and adults with ADHD the characteristics of sluggish cognitive tempo (SCT), a set of symptoms including slowed cognitive processing, confusion, lethargy, and apathetic behavior (6) that impact on social, emotional, and academic functioning. In *Neuropsychological correlates of ADHD: indicators of different attentional profiles among youth with sluggish cognitive tempo*, Krone et al. used the well-validated objective assessment measures the Attention Network Test (ANT) and the Continuous Performance Test (CPT) to evaluate different aspects of attention in youth with ADHD and typically developing controls. The authors detected a partial neurocognitive independence of SCT from ADHD, since inattentive symptoms of ADHD were related to cognitive and behavioral control measures on the CPT, whereas SCT symptom severity was related to the alerting/arousal measure on the ANT. Results from this investigation provides additional support for the validity of SCT as a clinically meaningful construct separate from ADHD-inattention symptoms, paving the way for ad-hoc treatments. This finding was confirmed in *Characteristics of Sluggish Cognitive Tempo among adults with ADHD: objective neurocognitive measures align with self-report of executive function* by the same group, Krone et al., who investigated the clinical and neuropsychological characteristics of SCT in a cohort of adults with ADHD. Specifically, they highlighted that adults with ADHD and SCT showed a distinct profile characterized by deficits in executive functions and symptoms of emotional dyscontrol. Moreover, both clinician and self-report consistently detected a significantly greater impairment in individuals with ADHD plus SCT compared to controls with ADHD only, providing useful insights for both evaluation and treatment procedures.

Dimitri et al. in *Observing the behavioural effects of methylphenidate in children and adolescents with ASD-ADHD dual diagnosis: A mini review* reviewed the literature concerning the behavioural effects of methylphenidate in youth with a dual diagnosis of ASD and ADHD. This rigorous systematic mini review concluded that methylphenidate is currently the most suitable drug for the treatment of hyperactivity and inattention in youth with both ASD and ADHD, but future randomized controlled trials should be conducted in order to establish with appropriate evaluation methods the most effective drug treatments for these complex patients.

Finally, Pomè et al. in their investigation *Autistic individuals show less grouping-induced bias in numerosity judgments* used a psychophysiological paradigm to assess possible differences between ASD individuals and control peers in perceived numerosity of grouped objects. They found that numerosity estimation was reduced only in control individuals when were proposed connected dots grouped into a single item, suggesting different perceptual strategies in ASD subjects. Therefore, the results support autistic differences in global vs. local processing already proposed in the weak central coherence theory (7), and in the enhanced perceptual functioning account (8).

To conclude, this Research Topic has produced different relevant papers that allow researchers to deepen their understanding of NDDs from multiple levels of analysis. Additionaly, since NDDs are the most prevalent chronic medical conditions viewed in pediatric primary health care (9), these papers could also help clinicians in their decision making and treatment choices.

As Editors, we would like to thank all the authors for their valuable contributions, the reviewers for their time in critically reviewing the papers and posing insightful comments, and the editorial team of the journal Frontiers in Child and Adolescent Psychiatry for their collective support and constant assistance.

## References

[1] American Psychiatric Association. Diagnostic and Statistical Manual of Mental Disorders. Arlington, VA: American Psychiatric Association Publishing (2013).

[2] Francés L Quintero J Fernández A Ruiz A Caules J Fillon G Current state of knowledge on the prevalence of neurodevelopmental disorders in childhood according to the DSM-5: a systematic review in accordance with the PRISMA criteria. Child Adolesc Psychiatry Ment Health. (2022) 16(1):27. 10.1186/s13034-022-00462-1 35361232 PMC8973738

[3] Jensen M Girirajan S. Mapping a shared genetic basis for neurodevelopmental disorders. Genome Med. (2017) 9:109. 10.1186/s13073-017-0503-4 29241461 PMC5729609

[4] Shaw P Gogtay N Rapoport J. Childhood psychiatric disorders as anomalies in neurodevelopmental trajectories. Hum Brain Mapp. (2010) 31:917–25. 10.1002/hbm.21028 20496382 PMC6870870

[5] Hansen BH Oerbeck B Skirbekk B Petrovski BÉ Kristensen H. Neurodevelopmental disorders: prevalence and comorbidity in children referred to mental health services. Nord J Psychiatry. (2018) 72(4):285–91. 10.1080/08039488.2018.1444087 29488416

[6] Becker SP Leopold DR Burns GL Jarrett MA Langberg JM Marshall SA The internal, external, and diagnostic validity of sluggish cognitive tempo: a meta-analysis and critical review. J Am Acad Child Adolesc Psychiatry. (2016) 55(3):163–78. 10.1016/j.jaac.2015.12.006 26903250 PMC4764798

[7] Happé F Frith U. The weak coherence account: detail focused cognitive style in autism spectrum disorders. J Autism Dev Disord. (2006) 36(1):5–25. 10.1007/s10803-005-0039-0 16450045

[8] Mottron L Dawson M Soulières I Hubert B Burack J. Enhanced perceptual functioning in autism: an update, and eight principles of autistic perception. J Autism Dev Disord. (2006). 10.1007/s10803-005-0040-7 16453071

[9] Zablotsky B Black LI Maenner MJ Schieve LA Danielson ML Bitsko RH Prevalence and trends of developmental disabilities among children in the United States: 2009–2017. Pediatrics. (2019) 144(4):e20190811. 10.1542/peds.2019-0811 31558576 PMC7076808

